# Cardiometabolic Risk in Acromegaly: A Review With a Focus on Pasireotide

**DOI:** 10.3389/fendo.2020.00028

**Published:** 2020-02-06

**Authors:** Soraya Puglisi, Francesco Ferraù, Marta Ragonese, Federica Spagnolo, Salvatore Cannavò

**Affiliations:** ^1^Endocrine Unit, University Hospital G. Martino, Messina, Italy; ^2^Internal Medicine, Department of Clinical and Biological Sciences, University of Turin, Turin, Italy; ^3^Department of Human Pathology of Adulthood and Childhood, University of Messina, Messina, Italy

**Keywords:** pasireotide, acromegaly, GH-secreting pituitary tumor, acromegaly treatment, cardiometabolic risk

## Abstract

Acromegaly is a disease due to chronic GH excess and a consequent rise in IGF-1 levels. This rare endocrine condition is associated with metabolic alterations such as hyperglycaemia, dyslipidaemia, and systemic arterial hypertension, which, in addition to GH excess-related cardiovascular changes, play critical roles in increasing cardiovascular risk and mortality rates. Biochemical control of acromegaly, achieved by means of surgical, and/or medical treatment, positively impacts on cardiovascular risk factors and metabolic alterations, reducing overall patient mortality. However, treatment modalities of acromegaly and disease control differently impact on glucose homeostasis and lipid changes, and consequently on cardiometabolic risk. In this regard, pasireotide was shown to significantly influence glucose metabolism. This review summarizes the cardiometabolic consequences of acromegaly and its treatment, focusing on available data around the effects of medical therapy with pasireotide on factors that influence cardiometabolic risk.

## Cardiovascular and Metabolic Complications in Acromegaly: an Overview

Acromegaly is a rare disease, resulting from excessive GH production and a consequent rise of IGF-1 levels. This condition is associated with increased morbidity and mortality when compared with an age-matched general population, mainly owing to higher cardiovascular risk and a worse metabolic profile (1, 2). In fact, cardiovascular disease seems to contribute toward death in 23–50% of cases (depending on patient age, or years in which the studies were conducted) (3). It is known that increased levels of GH/IGF-1 have a sodium-retaining effect, causing an increase in extracellular volume which contributes to hypertension (4), as well as determining morpho-functional heart alterations such as hypertrophy, diastolic dysfunction, up to systolic dysfunction in the end-stage (5).

Hypertension affects 33.6% of patients with acromegaly, ranging from 11 to 54.7% in different series (6), and represents an independent predictor of mortality (7). A positive correlation between disease duration and the prevalence of hypertension (8) has been demonstrated, similar to what is observed about other cardiac complications in acromegaly patients, including valvulopathy (found, at diagnosis, in ~75% of patients), and arrhythmias (occurring ~90% of patients with active disease) (3). Conversely, conflicting data has been reported regarding the prevalence of atherosclerosis in acromegaly, since some authors reported increased incidence of carotid intima-media thickness (9), while others did not confirm this finding (10). Similarly, one study failed to demonstrate an increased risk for coronary artery disease (11), while we found, by means of an integrated assessment of the Framingham score with the Agatston coronary artery calcium score, a risk of coronary atherosclerosis in 40% of acromegaly patients, and evidence of coronary calcifications in about half of them (12). Moreover, a subsequent study of our group demonstrated reduced life expectancy in acromegaly patients with a high Framingham score, and an increased risk of lethal ischemic cardiovascular (CV) events associated with an elevated Agatston score (13).

In this context, the role of lipid abnormalities in acromegaly is crucial. It is known that GH/IGF-I hypersecretion provides anabolic and lipolytic effects, while the most common lipid profile alterations are hypertriglyceridemia (occurring in 33–40% of patients with acromegaly, which is three times higher than in the general population) and low levels of HDL cholesterol (39–47% of cases), whereas the prevalence of hypercholesterolemia is similar to that of the general population (3). On the other hand, circulating levels of Lp-a, Apo A-I, and Apo E, which are involved in the transport of triglycerides, cholesterol, and small dense LDL particles, and promote the development of vascular damage and atherosclerosis, were found to be elevated in acromegaly patients (3).

In addition, a notable contribution to the unfavorable cardiovascular profile of acromegaly patients is provided by glucose metabolism impairment. GH/IGF-1 hypersecretion affects insulin sensitivity, promotes gluconeogenesis, reduces glucose uptake in fat tissue and muscles, and impairs the function of β pancreatic cells (14). Consequently, the prevalence of glucose metabolism abnormalities in acromegaly exceeds that of the general population, varying significantly between studies mainly due to the heterogeneity of diagnostic criteria. The frequency of diabetes mellitus (DM) ranges from 16 to 56%; impaired glucose tolerance (IGT) from 6 to 45%; and impaired fasting glucose (IFG) from 7 to 22% (3). In patients with acromegaly, DM occurs at a younger age than in the general population (15, 16), and is associated with a worse prognosis (16, 17). It is worthy of note that the development of glucose metabolism abnormalities is associated with disease duration and severity, but they often occur in the earliest phases of acromegaly (14). In some studies, DM has been found to correlate positively with hypertension and to be a predictor of disease activity, mortality, and of a low probability of achieving disease control (7, 15, 16).

Finally, acromegaly is associated with sleep apnea (occurring in 45–80% of cases, according to a different series) (18), which is known to be predisposing to hypertension and arrhythmias, as well as affect metabolic profiles, and therefore contribute to increased cardiovascular mortality.

## Effects of Biochemical Control of Acromegaly on Cardiometabolic Complications

In acromegaly, transsphenoidal surgical removal of the pituitary adenoma remains the treatment of choice in most cases, but medical therapy has been playing an increasing role in recent decades (19). First-line medical therapy consists of somatostatin analogs (SSA), including octreotide and lanreotide, with proven efficacy for around 50% of patients in terms of GH control and IGF-1 normalization (20). Nevertheless, encouraging data about the efficacy of pasireotide, a new multireceptor-targeted SSA, has been provided (21). Moreover, a significant figure (60–90%) of patients treated with the GH-receptor antagonist pegvisomant (PEG) achieve IGF-1 normalization (22–25). Further treatment options to help obtain disease control are cabergoline and radiotherapy (19).

The benefits of acromegaly control have been demonstrated at multiple levels. First of all, achieving biochemical control seems to positively impact on mortality risk. In 2004, Holdaway et al. reported a mortality rate reduced to near that of the general population among acromegaly patients who obtain normalization of serum IGF-1 and reduction of GH values <1 μg/L (7). The same study also showed that the last GH (or, in substitution, the last IGF-1) value was an independent predictor of survival in multivariate analysis. These findings were confirmed by meta-analysis, which reported a Standard Mortality Rate (SMR) close to that of the general population in biochemically controlled acromegaly patients (random serum GH <2.5 μg/L, or normalization of IGF-1 levels at the last follow-up) (26). Accordingly, a retrospective single-center study confirmed that SMR was higher in acromegaly patients when GH was >2.5 ng/ml at last visit, showing that overall mortality can be reduced by using a multimodal approach and carefully managing comorbidities (27). More recently, Postma et al. showed that SMR was not increased in adequately controlled acromegaly patients (28). Taking into account that one of the most important causes of death in acromegaly patients is CV events (7, 15, 29), it is reasonable to suppose an effect of acromegaly control on CV diseases. This hypothesis has been indirectly confirmed by Jayasena et al. (30), who demonstrated an association between ischemic heart disease and a high GH burden (calculated for each patient by multiplying the number of years of disease duration by the mean level of GH recorded during the entire follow-up), and between cerebrovascular disease/cardiomyopathy and a higher mean IGF-1 burden (calculated similarly to the GH burden, using the mean IGF-1 index). Similarly, Varadhan et al. (31) reported significantly higher cumulative GH exposure in acromegaly patients who died and in those who developed hypertension, diabetes, or CV events during follow-up. In contrast to these findings, a retrospective study, including 200 acromegaly patients (11.8% of whom reported CV events during follow-up), only identified age and smoking as predictors of CV events in multivariate analysis, although a lack of control of acromegaly was the strongest determinant of diabetes (32).

The effects of attaining control over GH and IGF-1 excess have been demonstrated by acromegalic cardiomyopathy. An improvement of the left ventricular mass index has been reported in patients treated with surgery (9, 33), SSA (33–35), and PEG (36). The effects of biochemical control of acromegaly on hypertension are more controversial, being heterogeneous across studies. Some authors reported a higher rate of hypertension in uncontrolled acromegaly than in patients with controlled disease (37, 38), while other researches resized the relevance of the normalization of hormonal parameters on hypertension (32, 39).

Moreover, blood pressure profile also seems to be influenced by abnormalities of glucose metabolism (40), thus adding a further incentive to control metabolic comorbidities. In this context, there are consistent data about the improvement of glycemic alterations after surgery (34, 41). A recent study, assessing 151 newly diagnosed acromegaly patients with an oral glucose tolerance test (OGTT) before and 3–12 months after surgery, showed an improvement of glucose tolerance in 87.3% patients with IGT, and in 66.7% of those with DM (41). Conversely, medical therapies exert different effects on glucose metabolism. Although octreotide and lanreotide reduce pancreatic insulin secretion, the negative impact on glucose homeostasis seems to be counterbalanced by the improvement of insulin resistance (14). However, the debate is still open, and several trials have been carried out to clarify this issue (Table 1). In the last decade, two meta-analyses led to different conclusions (Table 1) (42, 49). Mazziotti et al. (42), evaluating 31 retrospective and prospective studies performed from 1987 to 2008, concluded that conventional doses of octreotide and lanreotide do not significantly increase fasting plasma glucose (FPG) or serum glycosylated hemoglobin (HbA1c), while possibly negatively impacting on response to OGTT. Conversely, Cozzolino et al. (49) analyzed 47 prospective studies performed from 1993 to 2017 and showed an increase of HbA1c and glucose levels after OGTT, reporting a significant increase of FPG only if SSA were used as second-line treatment.

**Table 1:** Effects of first- and second-generation SSAs on glucose metabolism.

**References**	**Study design**	**Subjects**	**Follow up**	**Drug**	**FPG**	**FPI**	**HbA1c**	**IGR OGTT**
Mazziotti et al. (42)	Meta-analysis of 31 acromegaly studies published from 1987 to 2008.	624 AP	3 w−96 m	SSAs	NS	↓	NS	↑
Ghigo et al. (43)	Multicenter, randomized, open-label study with two parallel groups.	118 AP (57 vs. 56)	13 m	OCT-LAR	↑	↓	↑	↑
				PEG	↓	↓	↓	↓
Henry et al. (44)	Randomized, double-blind, single-center study.	45 HV	7 d	PAS s.c. bid	NS	↓		↑
Gadelha et al. (21)	Multicenter, randomized, phase 3 trial.	198 (130 vs. 68) AP	24 w	PAS-LAR vs. SSAs	↑		↑	
Colao et al. (45)	Multicenter, prospective, randomized, double-blind study.	358 (176 vs. 182) AP	12 m	PAS-LAR vs. OCT-LAR	↑		↑	
Breitschaft et al. (46)	Randomized, open-label, five-arm study (Pasireotide in combination with antihyperglycemic agents).	90 HV	7 d	PAS s.c. bid	↑	↓		↑
Bronstein et al. (47)	Crossover extension randomized, double-blind, multicenter phase III trial.	119 (81 vs. 38) AP	12 m	PAS-LAR vs. OCT-LAR	↑		↑	
Fleseriu et al. (48)	Multicenter, open-label, single arm, expanded-treatment protocol for Pasireotide long-acting.	44 AP	4–70 w	PAS-LAR	↑	NS	↑	
Cozzolino et al. (49)	Meta-analysis of 47 prospective interventional trials.	1297 AP	6–60 m	SSAs	NS	↓	↑	↑
Shimon et al. (50)	Retrospective multicenter study.	35 AP	at least 2 m	PAS-LAR	↑		NS	
Muhammad et al. (51)	Prospective, single-center, open-label, investigator-initiated conversion study.	61 AP	12 + 12 w	PAS-LAR±PEG vs. SSA+PEG	↑		↑	

*AP, Acromegaly patients; HV, Healthy volunteers; SSAs, somatostatin analogs; d, days; w, weeks; m, months; PAS-LAR, Pasireotide long acting; PEG, Pegvisomant; OCT-LAR, Octreotide long acting; FPG, fasting plasma glucose; FPI, fasting plasma insulin; HbA1c, glycosylated hemoglobin; IGR OGTT, Impairment of glucose response to oral glucose tolerance test*.

The effect of PEG on glycemic alterations is more defined, showing improvement of insulin sensitivity, FPG, and HbA1c in IGT and DM, although it is debated whether these changes are due to biochemical control (without inhibition of insulin secretion by SSA) or specific drug effects (43, 52, 53).

Biochemical control of acromegaly also impacts on lipid alterations. A retrospective study showed an improvement of hypertriglyceridemia and HDL cholesterol levels after normalization of IGF-1/GH levels achieved with either surgery or medical therapy, although this did not occur in all patients with controlled disease (54). More recently, 42 patients with untreated active acromegaly were prospectively studied, evaluating changes in metabolic profile after surgery and comparing those who achieved remission with those who did not. Total and LDL cholesterol levels did not change after surgery for either controlled or uncontrolled patients. Conversely, in the remission group, HDL levels rose while triglycerides levels fell, whereas in patients with persistent active acromegaly there was no change (55). A 48-week treatment with lanreotide autogel (120 mg/4 weeks) has been reported to significantly decrease triglycerides and increase HDL cholesterol levels in non-diabetic acromegaly patients, without significant changes to total as well as LDL cholesterol levels (56).

Finally, medical therapy of acromegaly improves sleep apnea, although frequently irreversible alterations of the craniofacial anatomy and the upper respiratory tract hamper a total remission (18). The beneficial effects of SSA are due to the reduction in swelling of the upper airway soft tissue, and particularly of tongue volume (57). Grunstein et al. (58) reported a 40% decrease in total apnea time, with an improvement in indices of oxygen desaturation and sleep quality after 6 months of octreotide. However, the authors showed no correlation between the decrease in total apnea time and the reduction of GH levels. Similarly, Annamalai et al. (59) demonstrated an improvement of the apnea/hypopnea index score in 61% of patients treated for 24 weeks with lanreotide autogel, but without any correlation to changes of GH and IGF-1. Therefore, medical treatment with SSA reduces the severity of sleep apnea, which can persist despite normalization of GH levels or significantly improve, even in cases of partial biochemical remission.

## The Cardiometabolic Risk Among Acromegaly Patients Treated With Pasireotide

Treatment modalities of acromegaly and disease control impact differently on glucose homeostasis and lipid changes, and consequently on cardiometabolic risk. In the class of SSA, pasireotide (PAS) was shown to significantly influence glucose metabolism (60). PAS is a multi-receptor targeted SSA with higher binding affinity for somatostatin receptor (SSTR) 1, 3, and 5 and lower for SSTR2 than octreotide and lanreotide (61).

Clinical studies demonstrated that, compared to OCT-LAR, PAS long acting release (PAS-LAR) treatment was more effective in achieving biochemical control in acromegaly patients, and was able to provide greater biochemical control in patients inadequately controlled by OCT-LAR or lanreotide autogel (21, 45, 47, 62). Clinical trials demonstrated the efficacy but also the safety of treatment with PAS, although hyperglycaemia-related adverse events were common (63). Indeed, Colao et al. showed that hyperglycaemia occurred in the 28.7% of patients treated with PAS-LAR and in the 8.3% of those treated by OCT-LAR (45). Moreover, antidiabetic medication was needed in 44.4% of patients treated with PAS-LAR, and in 26.1% of those treated with OCT-LAR (45). In the PAOLA study, at 24-week evaluation, 21 and 26% of 198 patients treated with PAS-LAR 40 mg/ or 60 mg/28 days, respectively, developed diabetes mellitus (21). In a 12-month crossover extension of the PAOLA study, patients with inadequate biochemical control at the end of core study were switched to PAS-LAR or OCT-LAR. In the PAS-LAR treated group, 13.6% discontinued the treatment because of hyperglycaemia-related adverse events, while no one discontinued OCT-LAR because of glucose metabolism alterations (47). Results from the ACCESS study documented hyperglycaemia-related adverse events in 45.5% of acromegaly patients receiving PAS-LAR 40 mg/28 days, with type 2 diabetes mellitus occurring in 13.6% of cases. Mean HbA1c and fasting plasma glucose levels significantly increased, respectively, from 5.9% and 100.4 mg/dl at baseline to 6.8% and 135.9 mg/dl after 3 months of treatment (48). Recently, in a “real life” retrospective multicentre study, PAS-LAR was shown to be effective in 54% of acromegaly patients resistant to OCT-LAR or lanreotide autogel, and treated with PAS-LAR, while 63% experienced glucose metabolism alterations, leading to initiation or intensification of antidiabetic treatment in 77.3% of them (50).

In the PAPE study, 61 patients with controlled acromegaly under SSA or PEG were switched to PAS-LAR with or without PEG. After 24 weeks of treatment, PAS-LAR was well-tolerated, but the 88.5% of adverse events were hyperglycaemia-related (65.6% were grade 1 and 2, while 23.0% were grade 3 and 4; all related to PAS-LAR). Moreover, the number of diabetic patients doubled, increasing from 32.8% at baseline to 68.9% at the end of the study. After 24 weeks, 44% of patients started an antidiabetic treatment while almost 25% had to change to a glucose-lowering drug, most of them treated with a combination of metformin and DPP4 inhibitor (51). In the PAPE extension study, frequency of diabetes mellitus increased from 68% at 24 weeks to 77% at 48 weeks, while 9 patients had to discontinue PAS-LAR because of severe hyperglycaemia. In this study, moreover, PAS-induced hyperglycaemia was inversely correlated with baseline insulin secretion. In this regard, in clinical trials, the risk of developing hyperglycaemia was higher in patients who were defined diabetic or prediabetic at baseline (51). In the PAOLA study, 31 and 26% of the patients receiving PAS-LAR 40 mg/ and 60 mg/28 days, respectively, were treated with antidiabetic medication at study entry, while 25% in each group initiated antidiabetic drugs during the study. Patients who received antidiabetic medications during PAS-LAR treatment had higher baseline mean FPG and HbA1c levels than patients who did not require treatment for diabetes. In detail, among patients with baseline FPG >100 mg/dL, 52 and 71% of those receiving PAS-LAR 40 or 60 mg respectively, developed hyperglycaemia during treatment (21). Overall these findings would suggest that baseline glucose status is a potential predictive factor for the development of hyperglycaemia during PAS-LAR treatment. Therefore, before starting PAS treatment, patients should undergo a glucose metabolism evaluation, and in case of glycaemic abnormalities, antidiabetic medication should be optimized before starting PAS therapy.

Response to PAS-LAR treatment does not seem to influence glucose homeostasis alterations, since the effect on HbA1c was similar for responders and non-responders in the PAOLA study (21). Interestingly, in the same study, PAS-associated elevations in glucose levels generally plateaued after ~2–3 months of treatment, as already reported. The hyperglycaemic effect of PAS can be explained by its binding affinity to SSTR. Indeed, glucagon is produced by cells expressing SSTR2, while insulin is produced by cells that also express SSTR5. Due to the high binding affinity to SSTR5, PAS suppresses insulin secretion, while it does not induce an effective inhibition on glucagon secretion. Furthermore, PAS appears to reduce glucagon-like peptide 1 (GLP-1) and glucose-dependent insulinotropic polypeptide (GIP) levels, while leaving insulin sensitivity unaffected (44). Vildagliptin and liraglutide were the most effective drugs in minimizing PAS-related glucose alterations in healthy volunteers, confirming the existence of an anomalous incretin response (46).

However, available data on PAS treatment overall suggests a generally reversible hyperglycaemic effect which requires active monitoring and is manageable with an appropriate therapeutic strategy involving available antidiabetics agents. On the other hand, the adverse metabolic effects of PAS can impact negatively on the cost-effectiveness of this treatment when compared with PEG, considering the costs related to the management of hyperglycemia-related adverse events (64).

The effect of PAS on lipid profiles has been investigated by Chen et al. who studied pharmacokinetics and safety of PAS in 45 healthy Chinese male subjects (65), showing that PAS reduced serum cholesterol and triglyceride levels in a dose-dependent manner.

In this regard, it is worth of mentioning that lipid profile influences the visceral adiposity index (VAI) that is a marker of visceral fat dysfunction, indirectly reflecting cardiometabolic risk. In acromegaly, VAI seems to be strongly related to insulin resistance, adipose tissue dysfunction, and cardiometabolic risk, especially in post-menopausal women (66). Interestingly, some authors demonstrated that 1 year of PAS treatment had a positive effect on cardiometabolic risk in 16 patients with Cushing's disease (CD), despite the increase of diabetes mellitus prevalence (67). Indeed, PAS treatment improved anthropometric parameters, reduced the prevalence of dyslipidaemia, ameliorated the lipid profile, and significantly reduced VAI and adipose tissue dysfunction in CD patients (67). However, studies of acromegaly patients which address the impact of PAS treatment on lipid changes and adipose tissue dysfunction as factors influencing cardiometabolic risk, regardless the effects on glucose homeostasis, are needed.

In conclusion, acromegaly is associated with metabolic alterations, such as hyperglycaemia, dyslipidaemia, and systemic arterial hypertension, which in addition to cardiovascular changes play a critical role in increasing cardiovascular risk and mortality rate. Surgical or medical control of acromegaly, with SSA or pegvisomant, positively influences cardiovascular risk factors, metabolic alterations, and related mortality. Pasireotide-related beneficial effects in terms of biochemical and clinical disease control can be burdened by its diabetogenic effect, but can be adequately counteracted by a proper pharmacological strategy, while—in this context—studies on other cardiometabolic outcomes are still needed.

## Author Contributions

SP, FF, MR, and FS reviewed the literature. SP, FF, and MR wrote the manuscript. SC revised the manuscript.

### Conflict of Interest

The authors declare that the research was conducted in the absence of any commercial or financial relationships that could be construed as a potential conflict of interest.
